# Resonance energy transfer in orthogonally arranged chromophores: a question of molecular representation†

**DOI:** 10.1039/d4cp00420e

**Published:** 2024-04-04

**Authors:** Richard Jacobi, Leticia González

**Affiliations:** a Doctoral School in Chemistry (DoSChem), University of Vienna, Währinger Straße 42 1090 Vienna Austria; b Vienna Research Platform on Accelerating Photoreaction Discovery, University of Vienna, Währinger Straße 17 1090 Vienna Austria leticia.gonzalez@univie.ac.at +43 1 4277 52750; c Institute of Theoretical Chemistry, Faculty of Chemistry, University of Vienna Währinger Straße 17 1090 Vienna Austria

## Abstract

Energy transfer between orthogonally arranged chromophores is typically considered impossible according to conventional Förster resonance energy transfer theory. Nevertheless, the disruption of orthogonality by nuclear vibrations can enable energy transfer, what has prompted the necessity for formal expansions of the standard theory. Here, we propose that there is no need to extend conventional Förster theory in such cases. Instead, a more accurate representation of the chromophores is required. Through calculations of the energy transfer rate using structures from a thermal ensemble, rather than relying on equilibrium geometries, we show that the standard Förster resonance energy transfer theory is still capable of describing energy transfer in orthogonally arranged systems. Our calculations explain how thermal vibrations influence the electronic properties of the states involved in energy transfer, affecting the alignment of transition dipole moments and the intensity of transitions.

## Abstract 2

Through calculations of the energy transfer rate using structures from a thermal ensemble, we show that the standard Förster resonance energy transfer theory is capable of describing energy transfer in orthogonally arranged systems.

## Graphical abstract

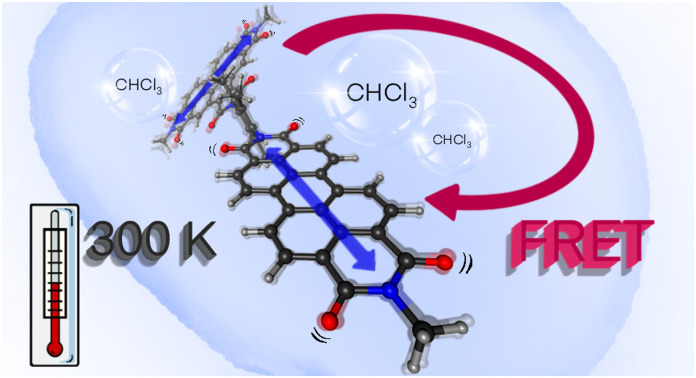

## 1 Introduction

**Fig. 1:**
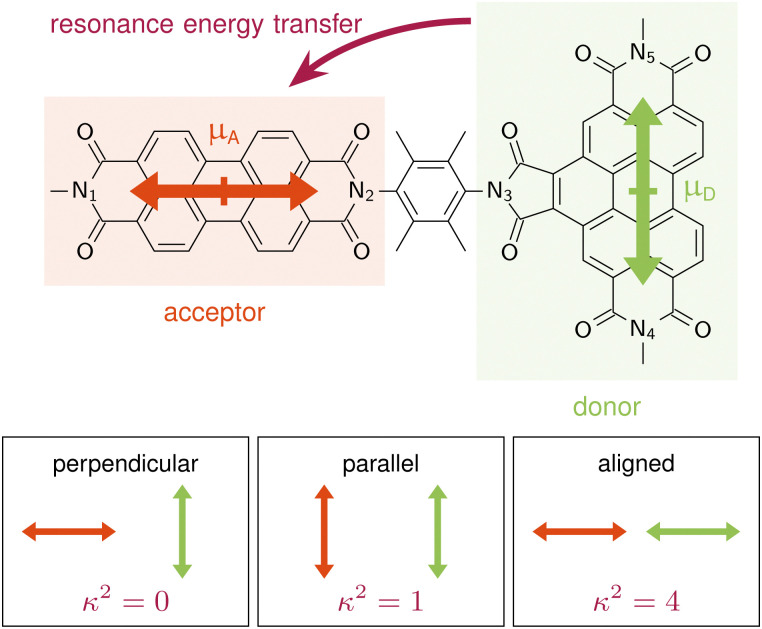
Benzoperylene-diimide perylene-diimide dyad (BPDI-PDI), where the TDM of the donor is arranged orthogonally to the TDM of the acceptor and the distance vector. Pictograms on the bottom highlight the orientation factor *κ*^2^ according to standard RET theory for distinct TDM conformations.

Förster's theory on resonance energy transfer (RET)^1–3^ is a useful tool to describe excitation energy transfer processes. RET plays a key role in energy harvesting^4–7^ and can be used to investigate conformations,^8–10^ interactions^11–14^ and dynamics^15^ on a molecular level. The central merit of this theory is the formalization of the energy transfer as a coulombic dipole–dipole resonance of the transition dipole moments (TDMs) of a respective donor (D) and acceptor (A) chromophore. These coulombic interactions are collected in the electrostatic interaction factor |*V*_DA_|^2^,1
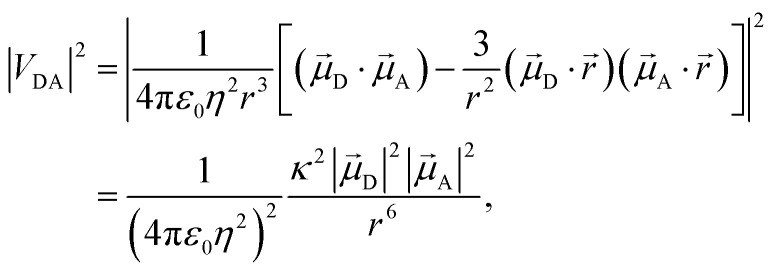
where *μ⃗*_D_ and *μ⃗*_A_ are the TDMs of the donor and acceptor chromophores, respectively, *r⃑* is the distance vector with its magnitude *r*, and *ε*_0_ and *η* are the vacuum permittivity and medium refractive index, respectively. In the second part of the equation, the alignment dependence of the dot products between the TDMs and the distance vector are collected in the orientation factor *κ*^2^. This factor can take on values between 0 for orthogonal TDM arrangements and 4, when the TDMs are both aligned with *r⃑*, see Fig. 1.^16,17^ The total RET rate then depends on both |*V*_DA_|^2^ and the spectral overlap *J* of the donor fluorescence and acceptor absorption spectra, both normalized to the unit area,2
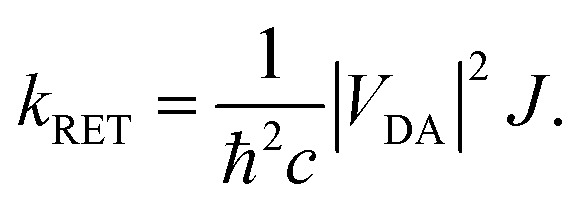


Langhals and co-workers challenged this theory by synthesising a benzoperylene-diimide perylene-diimide dyad (BPDI-PDI), where two chromophores are linked with a stiff spacer to enforce orthogonality of the TDMs (Fig. 1).^18^ Intriguingly, despite the orthogonal arrangement, the emission of the donor was completely quenched in the dyad by efficient energy transfer occurring at a time constant of 9.2 ps.^19^ Investigations of the energy transfer dependence on the stiffness of the linker^19,20^ and on the distance between the chromophores^21^ continued to question standard RET theory, suggesting a distance dependence that slightly differs from the *r*^6^ of eqn (1).^22^

A normal mode analysis of BPDI-PDI^19^ identified low-frequency vibrations that break the orthogonality of the TDMs and thus enable energy transfer. Such study inspired formal expansions of standard RET theory, aimed to incorporate vibrational effects by accounting for excitonic coupling fluctuations resulting from geometric distortions along the normal modes, at first solely with respect to the TDM arrangement,^22^ and later also on an energetic basis, where the energy levels are also influenced by the normal mode distortion.^23^ Even if such normal mode analyses neglect anharmonicities in the potential energy surface,^24^ the theoretical extensions rationalized why energy transfer is possible in orthogonally arranged chromophores such as the BPDI-PDI dyad,^18^ provided insight on how solvent environments influence the energy transfer efficiency^23^ and justified why the distance dependence is different from *r*^6^.^22^

In this paper, we demonstrate that formal extensions of Förster's theory are not required to explain RET in orthogonally arranged chromophores. We present an alternative perspective on how standard RET theory can account for energy transfer even when the equilibrium structure is unfavorable for RET efficiency: rather than altering the foundational theory, we depart from a frozen equilibrium structure and instead use an nuclear ensemble of structures that accurately represents the dynamic movement and distortions of the geometry at ambient temperature. We thus demonstrate that conventional RET theory is capable of accurately predicting the energy transfer rate in BPDI-PDI when one uses a thermal nuclear ensemble for the analysis and computes electronic properties directly on these representative structures. To this end, we investigate the photophysical properties of the molecular dyad BPDI-PDI both on the frozen equilibrium structure as well as on a thermal ensemble generated with molecular dynamics (MD) simulations.

## 2 Results and discussion

**Fig. 2:**
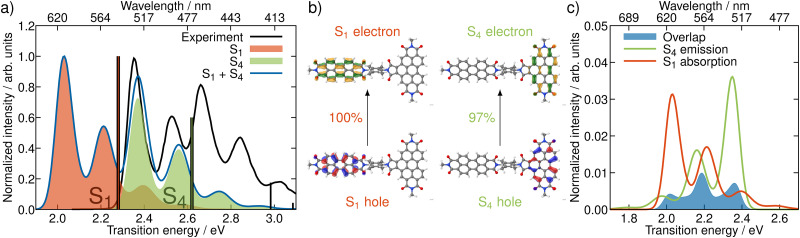
Properties of BPDI-PDI related to light absorption and emission. (a) Experimental (black) and vibrationally resolved spectrum computed on the frozen equilibrium structure (blue) of BPDI-PDI. The latter is computed as the sum of the vibrationally resolved spectra for the excitation to S_1_ (orange shaded area) and to S_4_ (green). Vertical transitions are shown as stick spectra. (b) NTOs for the excitations to S_1_ and S_4_. (c) Computed spectral overlap (blue area) between the emission from S_4_ (green) and the absorption to S_1_ (orange), both normalized to the unit area. The overlap intensity is enhanced compared to the spectra by a factor of 50 for better visibility.

In order to get an initial understanding of the photophysical properties of BPDI-PDI, we start by using time-dependent density functional theory to calculate vertical excitations on the frozen equilibrium structure of BPDI-PDI, *i.e.* on the optimized minimum energy structure. We truncate the aliphatic tails present in the experimental structure^18^ to methyl groups (as shown in Fig. 1), as the aliphatic tails were added in the experiment to improve the solubility, but do not influence photophysical properties and thus do not affect intramolecular energy transfer efficiency. The computational protocol on the frozen equilibrium geometry is explained in detail in Section S1 of the ESI.† As seen in Fig. 2a (stick spectra), the lower energy region of the spectrum until 3 eV is dominated by the S_1_ and S_4_ singlet excited states. However, perylene diimides usually exhibit a fine-structure in the absorption spectrum that is not properly recovered by simply convoluting purely electronic excitations.^16^ Therefore, and in order to reproduce the experimental signature accurately, we compute vibrationally resolved spectra using the Franck–Condon Herzberg–Teller method developed by Barone and co-workers.^25,26^ This approach computes the nuclear wavefunctions within the harmonic approximation as well as the change of TDMs with respect to the normal coordinates of the molecule in a first-order approximation to account for symmetry-allowed and symmetry-forbidden transitions. As this computation is performed for the pair of states involved in the electronic transition, we compute the vibrationally resolved spectra for the excitation to the S_1_ (orange shaded area in Fig. 2a) and to the S_4_ (green shaded area) electronic states. The full, vibrationally resolved absorption spectrum of BPDI-PDI in the region under 3 eV is then computed as the sum of the individual spectra (blue line in Fig. 2a).

We see that each individual spectrum for the excitation to the S_1_ or to the S_4_ resembles the three-peaked structure characteristic of perylene-diimides.^16^ Further, the full spectrum for the entire molecule reproduces well the experimental one,^18^ albeit red-shifted by around 0.4 eV. Since previous benchmarks for perylene-diimide based molecules have shown that 0–0 energies can vary between different functionals by more than 0.6 eV,^27^ our shift can be considered reasonable within the expected range. The good qualitative agreement between the spectral shape in experiment and computation, as well as the accurate reproduction of the relative energy between the individual peaks gives us the confidence that our time-dependent density functional theory setup is adequate for the description of the system.

The natural transition orbitals (NTOs) associated with the excitations to S_1_ and S_4_ reveal that both transitions are of ππ* character (Fig. 2b), but the S_1_ is localized in the acceptor moiety, while the S_4_ is in the donor. Since the NTOs are restricted to one fragment for each electron–hole pair, it appears plausible that the absorptive properties of the two fragments, which are linked together in the dyad, are not significantly affected by one another. This finding is in line with the experimental report,^18^ that the absorption spectra of the acceptor and donor chromophores in the dyad are additive. In fact, the computed vibrationally resolved spectra for the excitation to S_4_ and S_1_, respectively, resemble the experimental spectra of the isolated donor and acceptor chromophores. The sum of the individually computed spectra matches the experimental spectrum of the dyad. In contrast to this additive nature of the absorption, the fluorescence from the donor fragment is quenched in the experiment.^18^ As the S_4_ state is localized exclusively on the donor fragment, we identify this as the energy donating state. Consequently, S_1_ is the energy accepting state, which is localized solely on the acceptor fragment. As there is no wave function overlap between the two states represented by their NTOs (recall Fig. 2b), it is unlikely that energy transfer pathways requiring a wave function overlap, such as Dexter type energy transfer,^28^ which might compete with RET, play a significant role here. Additionally, Dexter type energy transfer has been excluded based on experimental data,^22^ supporting our observation. Therefore, we exclude energy transfer pathways other than RET from the analysis.

Now that we have identified the energy donating and accepting state, we can proceed to compute the properties associated with the energy transfer between them. *A priori*, it is not apparent why energy transfer should occur intramolecularly, especially with orthogonally-arranged chromophores. In principle, the energy could also be transferred from a BPDI-PDI molecule in the excited state to a second in the ground state, thus not limited by the intramolecular connectivity of the two chromophores. The possibility for intermolecular energy transfer is taken into account by performing an MD simulation of two dyads (Section S2, ESI†), where this time we also include the solubilizing alkyl tails present in the experiment.^18^ The results (Section S3, ESI†) evidence that the two dyads diverge rapidly and do not rejoin during the 250 ns simulation time. Based on this observation, we deem intermolecular energy transfer not important and henceforth focus on intramolecular RET.

According to RET theory (eqn (2)), energy transfer requires transitions to be resonant, *i.e.* the excitation energy of the emissive and absorptive states need to match. This can be quantified *via* the spectral overlap *J*, which is displayed between the normalized spectra for the emission from the S_4_ and the absorption to the S_1_ in Fig. 2c. There is a good overlap between the two spectra, resulting in a spectral overlap of *J* = 7.44 × 10^−3^ cm.

Besides the energetic requirement, efficient RET requires TDMs to be properly aligned. In our computations on the frozen equilibrium geometry, the TDMs of donor, *μ⃗*_D_, and acceptor, *μ⃗*_A_, are orthogonally aligned (89.94°), as targeted synthetically.^18^ Furthermore, *μ⃗*_A_ is parallel to the distance vector between the fragments, with *μ⃗*_D_ being perpendicular to the distance vector, as shown schematically in Fig. 1. This distance vector connects the centers of the fragments, which we define as the midpoint between the Nitrogen atoms, N_1_–N_2_ for the acceptor and N_4_–N_5_ for the donor. From eqn (1), it is clear that this TDM arrangement in the frozen equilibrium structure results in a *κ*^2^ value of 0 that impedes the possibility of energy transfer. Thus, we confirm that when using a frozen geometry, RET theory is insufficient to explain the efficient quenching of the donor emission.^18^

**Fig. 3:**
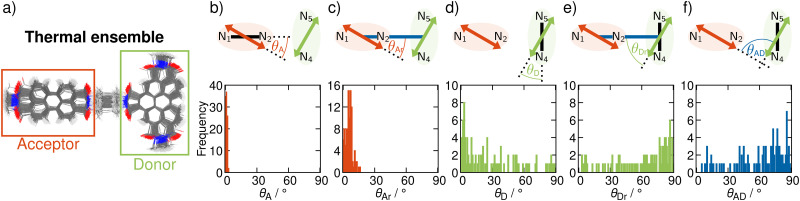
(a) 100 structures composing the thermal ensemble. (b)–(f) Histograms of the angles effecting the RET rate. Pictograms visualize the respective angles, where the TDMs of donor and acceptor are shown with green and orange arrows, respectively, the vectors connecting the nitrogen atoms of each fragment are represented as solid black lines, the distance vector between donor and acceptor is represented as a blue line. See the main text for more details.

In order to include nuclear vibrations in our model, we generate a thermal ensemble of 100 structures from MD simulations (Section S4, ESI†). Fig. 3a shows these 100 structures graphically overlaid on top of each other. For every structure, we then compute the TDMs of the donating and the accepting states, their magnitude and orientation, in order to unravel the electrostatic interaction factor |*V*_DA_|^2^ (eqn (1)) that properly accounts for the dynamic movement of the molecule. To elucidate the geometrical properties influencing |*V*_DA_|^2^, we analyse representative angles in Fig. 3. *θ*_A_ (panel b) is the angle between the acceptor TDM *μ⃗*_A_ and the N_1_–N_2_ vector. As this angle is 0 in the frozen equilibrium, it serves as a quantity to assess the deviation from the optimized structure within the ensemble. *θ*_Ar_ (panel c) is the angle between *μ⃗*_A_ and the distance vector *r⃑*, and directly influences |*V*_DA_|^2^. The analogous angles for the donor TDM (*θ*_D_ and *θ*_Dr_) are presented in panels d and e. Finally, panel f shows the angle between the TDMs, *θ*_AD_, which also directly influences |*V*_DA_|^2^.

As seen from Fig. 3b, *μ⃗*_A_ is well aligned with the N_1_–N_2_ vector, as *θ*_A_ is below 5° over the entire ensemble. This consistent alignment indicates that the electronic character of the accepting excited state does not change significantly with thermally induced nuclear motion. In contrast, the distribution of the angle between *μ⃗*_A_ and the distance vector *r⃑* is broader (Fig. 3c). This is a result of geometrical distortions being more pronounced for the whole dyad, as the entire molecule is more flexible than only one fragment alone. Nevertheless, given that the angles *θ*_Ar_ do not exceed 16°, *μ⃗*_A_ and *r⃑* can still be roughly considered as parallel.

**Fig. 4:**
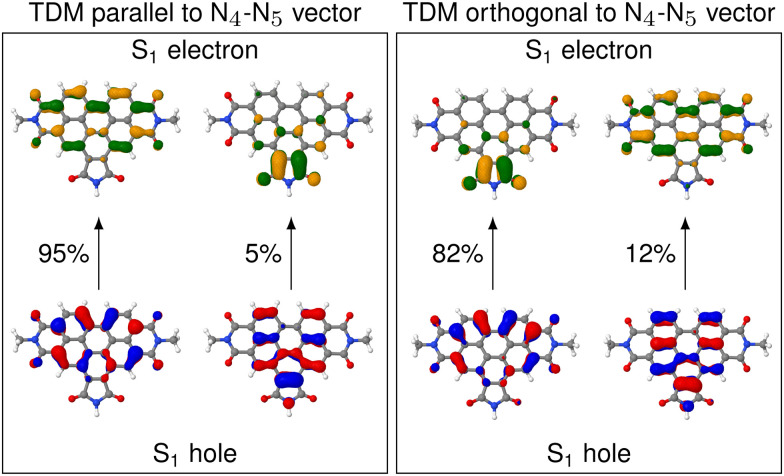
NTOs for the S_1_ excitations on the donor QM region (which corresponds to the S_4_ excitation in the full molecule) for two representative structures out of the ensemble. Left box shows the structure with the TDM aligned most parallel to the N_4_–N_5_ vector. Right box shows the same, but for the structure with the TDM aligned most perpendicular.

In contrast to *μ⃗*_A_, *μ⃗*_D_ is parallel to the N_4_–N_5_ vector only in a fraction of the nuclear ensemble (Fig. 3d). In fact, *θ*_D_ is larger than 20° in more than half of the structures. There are still some structures in which *μ⃗*_D_ is almost parallel to the N_4_–N_5_ vector, but in others *θ*_D_ is close to perpendicular. The high spread of angles can be attributed to the change in the character of the donating excited state as the molecule undergoes distortion. In fact, an analysis of the NTOs shows that the frontier orbitals for those structures, where *μ⃗*_D_ is parallel to the N_4_–N_5_ vector, are still of general ππ* character (see Fig. 4), similarly to what we observed in the frozen equilibrium structure (recall Fig. 2b). However, the areas, over which the excited electron is delocalized, are drastically different between structures where *μ⃗*_D_ is parallel to the N_4_–N_5_ vector and those, where it is perpendicular. While in the first case the density of the excited electron is mostly delocalized over the six-membered rings, it is to a large degree localized on the five-membered ring. Thus, the excitation to this state is of increased charge transfer character from the center of the donor fragment to the five-membered ring, to which the linker is attached. This charge transfer state is present in the frozen equilibrium structure as the S_7_ state, where about 50% of the excited electron is localized on the five-membered ring. It appears about 0.11 eV in energy above the donating state and is the next-higher state in energy, for which both the electron and hole are localized on the donor fragment. The presence of this state means that we deal with a pair of two diabatic states, which switch their energetic ordering depending on the structural distortions represented in the structures of the thermal ensemble, thus discriminating which of the two will be the donating state. This second donating state has not been part of the previously published investigations based on normal mode analyses, thus highlighting an advantage of our method.

The increased charge transfer character of the second state has two effects. On the one hand, the orientation of the TDM is changed from parallel to the N_4_–N_5_ vector to almost orthogonal to it, as can be seen from the angles. As described in more detail below, this improves the alignment of the TDMs in terms of RET efficiency. On the other hand, an increased charge transfer character results in a reduced intensity of the corresponding transition. This duality between improved alignment and reduced intensity will be focus of a more detailed analysis below. A more in-depth analysis of these NTOs can be found in Section S5 (ESI†).

The spread in alignment angles of the donor TDM can also be seen in the large range of angles between *μ⃗*_D_ and *r⃑* (*θ*_Dr_ in Fig. 3e). The trend here is approximately flipped, which means that there is a higher angle concentration close to 90°. This can be explained as follows: As seen from the pictograms in Fig. 3d and e, the distance vector *r⃑* (represented as a blue line) is mostly perpendicular to the N_4_–N_5_ vector. *θ*_D_ and *θ*_Dr_ both start at *μ⃗*_D_, but one goes to *r⃑*, the other one to the N_4_–N_5_ vector. As a consequence, *θ*_D_ and *θ*_Dr_ added for a single structure yield approximately the 90° representing the angle between *r⃑* and the N_4_–N_5_ vector. In other terms, whenever *θ*_D_ is close to 0°, *θ*_Dr_ is close to 90°, and *vice versa*.

The alignment of the acceptor and the donor TDMs as represented by the angles *θ*_Ar_ and *θ*_Dr_ give rise to the distribution of *θ*_AD_, the angle between *μ⃗*_A_ and *μ⃗*_D_ (Fig. 3f). Certainly, there are a lot of structures with arrangements close to orthogonal, as in the frozen minimum equilibrium geometry (these correspond to structures where *θ*_Ar_ is close to 0° and *θ*_Dr_ close to 90°). However, and most importantly, alignments away from perpendicularity are plenty – some even close to being parallel. The structures where *θ*_AD_ is close to 0 correspond to situations in which *μ⃗*_A_ points towards the acceptor fragment, thus aligning with *μ⃗*_D_. On average, *θ*_AD_ is around 61° in the ensemble. This is slightly different from the 72° measured with polarization-controlled transient absorption spectroscopy,^29^ which is due to the fact that a mere averaging does not include effects related to the intensity of the TDMs in the different structures. In other words, more intense snapshots contribute more to an effective angle than less intense ones. To account for the different intensities, we scaled each alignment angle by the product of TDM strengths |*μ⃗*_D_|^2^|*μ⃗*_A_|^2^ on a per-snapshot basis. The resulting scaled average amounts to around 74°, which accurately reproduces the experimentally reported angle.

**Fig. 5:**
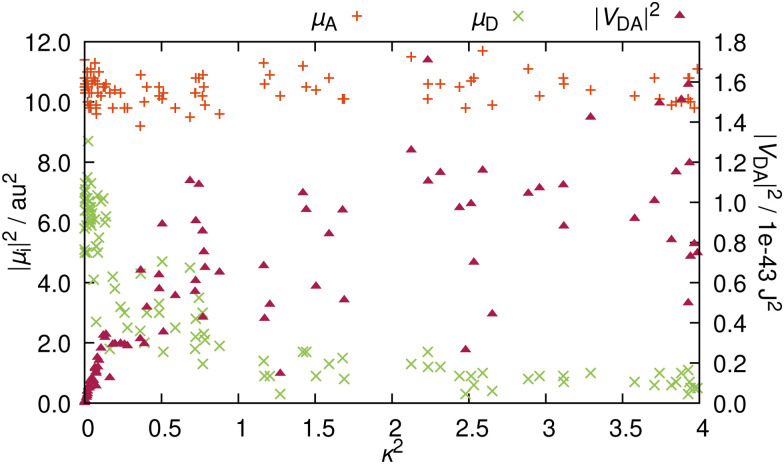
Influence of the RET orientation factor *κ*^2^ on the TDM strengths |*μ⃗*_A_|^2^ and |*μ⃗*_D_|^2^ as well as on the electrostatic interaction factor |*V*_DA_|^2^ for the 100 structures representative of the thermal ensemble.

It is now apparent that whenever *μ⃗*_A_ and *μ⃗*_D_ are not orthogonal to each other, RET is not geometrically forbidden. However, as pointed out above, the more favorable alignments come with an increased charge transfer character of the energy donating excited state. Therefore, as ππ* states usually exhibit large TDM magnitudes,^30^ it is questionable whether orientations that are more favorable for RET, come at the penalty of reduced intensities. In order to address this question, we compare the TDM strength, *i.e.* the transition intensity, to the RET alignment factor *κ*^2^ (Fig. 5). These two parameters strongly affect the electrostatic interaction factor |*V*_DA_|^2^.

The magnitude of *μ⃗*_A_ is independent from the resulting orientation factor – for each of the 100 structures, |*μ⃗*_A_|^2^ is between 9 and 12 a.u.^2^ (*ca.* 22 to 30 Debye). This behavior is as expected because the orientation of *μ⃗*_A_ is consistent for the entire ensemble, *i.e. μ⃗*_A_ does not significantly change with nuclear vibrations (recall Fig. 3b). In contrast, |*μ⃗*_D_|^2^ is well below |*μ⃗*_A_|^2^ (Fig. 5); only one outlier barely scratches 9 a.u.^2^, while the bulk exhibits TDM strengths of around 6 or 7 a.u.^2^. Notably, these intensities all occur at values for *κ*^2^ close to zero. With rising *κ*^2^, the intensities are quickly reduced, and for configurations yielding a *κ*^2^ of over 1, |*μ⃗*_D_|^2^ is consistently below 2 a.u.^2^ (about 5 Debye). This reduction in intensity reinforces the observation that the character of the excitation changes with the direction of *μ⃗*_D_. The change of the excited molecular orbital to become more localized on the five-membered ring does lead to a more favorable TDM alignment, but the increased charge transfer character of the excitation reduces the emission intensity, which causes this clearly visible inversely proportional relationship between |*μ⃗*_D_|^2^ and *κ*^2^.

In order to elucidate whether the reduction in RET efficiency due to the diminished TDM strength overweighs the improvement in RET efficiency caused by more favorable alignments, we compute the electrostatic interaction factor |*V*_DA_|^2^ for each structure. For this, in addition to the TDMs (orientation and magnitude), we need to compute the length of the distance vector, *r*. Details on how the fragment centers are defined can be found in Section S5 (ESI†). The resulting distances are between 16.7 and 17.3 Å, in line with previous descriptions at 17 Å.^19^ Additionally, the differences in distance within the ensemble are insignificant, so that for the analysis, the distance dependency of |*V*_DA_|^2^ is negligible. For RET, distances below 20 Å are very favorable, as RET is observed for distances well exceeding 50 Å.^17,31^ As the rate scales with *r*^6^ (recall eqn (1)), the energy transfer can be very efficient for such shorter distances.

The resulting values for |*V*_DA_|^2^ in relation to the *κ*^2^ values are also presented in Fig. 5. As *κ*^2^ enters eqn (1) as a factor, alignment factors close to zero naturally lead to an interaction factor close to zero. Upon a slight increase of *κ*^2^, the interaction factors instantly rise measurably. However, this direct proportionality levels off for *κ*^2^ over 0.5, where |*V*_DA_|^2^ is mostly independent of the orientation factor, and interaction factors at similar *κ*^2^ diverge by up to one order of magnitude. On the whole, gain in RET rate due to the more favorable alignments is mostly compensated by the loss in RET rate due to the reduced intensity, so that both these effects nullify each other, resulting in an averaged value for |*V*_DA_|^2^ of 5.2 × 10^−44^ J^2^ (*ca.* 0.20 eV or around 1580 cm^−1^).

This interaction factor can be combined with the spectral overlap to yield the energy transfer rate (eqn (2)). As stated elsewhere,^16^ it is not straightforwardly possible to compute vibrationally resolved spectra on a thermal ensemble generated from MD simulations. However, the vibrational fine structure is an important feature of the spectra for perylene-diimide derivatives, which is not properly accounted for by computing vertical excitation spectra on an ensemble. Therefore, we use the spectral overlap computed for the frozen equilibrium structure for the RET rate computation. These spectra are a reasonable representation of the energetic criteria for the RET efficiency, because they do include ensemble effects through the inclusion of vibrational transitions and the line broadening used for the convolution, even if they are not included on a per-structure basis. By combining the spectral overlap calculation from the frozen equilibrium structure with the electrostatic interaction factor computed on the thermal ensemble, we ensure a correct representation of the physical effects while avoiding the double-counting of any of these contributing effect.

Our computations yield a RET rate *k*_RET_ of 1.2 × 10^12^ s^−1^, which corresponds to a RET lifetime *τ*_RET_ = 1/*k*_RET_ of 0.86 ps (Section S6, ESI†). The calculated rate overestimates the experimentally^19^ recorded 1.06 × 10^11^ s^−1^ by about an order of magnitude. Nevertheless, for a theoretical estimation of an energy transfer rate, where small approximations can propagate and lead to massive errors, the results are in reasonably good agreement. The competing fluorescence lifetime is 6.8 ns.^19^ As this is about three to four orders of magnitude slower than the lifetime of the energy transfer, our results explain the efficient quenching of emission from the donor fragment.

## 3 Conclusion

In conclusion, we have demonstrated that the RET rate for the intramolecular energy transfer in BPDI-PDI can be accurately predicted from computations based on standard Förster's theory. Energy transfer in orthogonally-arranged chromophores is enabled by nuclear motion caused by the thermal energy available in the molecule. The idea that fluctuations or vibrations of the molecular structure lead to non-orthogonal arrangements has been already proposed in the literature.^18,19,22,23^ However, here we show that the effect of the thermal fluctuations of the dyad can be accounted for with classical molecular dynamics instead of considering low-frequency normal modes that need to be explicitly taken into account quantum mechanically and require extensions of Förster's theory.

Methodologically, we illustrate how important it is to go beyond the simple picture of a frozen equilibrium geometry and, instead, consider a nuclear ensemble, something which is rather usual when accurately computing absorption spectra. Here, MD simulations have been employed to generate such an ensemble, but other methods can be considered for this purpose and it would be interesting to see whether and how such nuances influence the results. In principle, our approach, *i.e.* to use classical molecular dynamics from where snapshots are selected to create a representative ensemble on which quantum calculations are done, is indeed a popular approach in computational spectroscopy or as a starting point for trajectory-based excited state dynamics, but we are not aware that it has been used to calculate RET rates of orthogonally-arranged dyes.

Our work on BPDI-PDI shows that the energy transfer does not only occur from one electronic state. Instead, we find that for different points in the ensemble, states of different electronic character take the donating role – a fact that has been neglected in existing approaches employing a normal mode analysis with only one reference electronic state. Extending this approach to other RET calculations could reveal electronic contributions to date ignored. Future work could also investigate whether the simple model presented here is also able to predict the energy transfer rates of BPDI-PDI derivatives,^19–21^ and explain whether thermally induced molecular distortions can also explain why the distance dependence diverged from the theoretical *r*^6^.

## Author contributions

L. G. conceived the work and acquired funding. R. J. designed the computational protocols, performed and analyzed all the calculations and wrote the first draft. L. G. supervised the calculations and contributed to the final version of the manuscript.

## Conflicts of interest

There are no conflicts to declare.

## Supplementary Material

CP-026-D4CP00420E-s001
