# Supplementary material for: Resonance energy transfer in orthogonally arranged chromophores: a question of molecular representation
Source: Phys Chem Chem Phys. 2024 Apr 4;26(16):12299–305. doi: 10.1039/d4cp00420e (PMC11041867; doi:10.1039/d4cp00420e)
Supplement: CP-026-D4CP00420E-s001 [file CP-026-D4CP00420E-s001.pdf]

Electronic Supplementary Material (ESI) for Physical Chemistry Chemical Physics.
This journal is © the Owner Societies 2024

Supporting Information to: Resonance energy transfer in orthogonally arranged chromophores: a question of molecular representation

Richard Jacobi and Leticia González

January 2024

Contents

S1 Computations Details at the Frozen Equilibrium Structure **S2**

S2 MD Simulation Protocol **S2**

S3 Role of Intermolecular Energy Transfer **S3**

S4 Computational Details at the Thermal Ensemble **S4**

S5 Character of the excitations in the ensemble **S8**

S6 Details on the RET rate computation **S10**

S1 Computations Details at the Frozen Equilibrium Structure

General setup

Photophysical properties are calculated with density functional theory (DFT) and its time-dependent version (TD-DFT) at the B3LYP/def2-SVP[1–4] level of theory, as implemented in the program package Gaussian 16, revision C.01[5]. In particular, the ground state geometries are optimized with DFT, and single point energies for the electronic excited states are obtained with TD-DFT over 30 singlets. Solvent effects for chloroform (as used in the experimental work[6, 7]) are included implicitly using the polarizable conductor-like calculation model[8, 9] by placing the solute in a cavity within the solvent reaction field. Dispersion interaction effects are corrected empirically using Grimme's D3 model with Becke-Johnson damping[10, 11]. The geometry optimization is performed with tighter cutoffs on forces and step size (`tight` keyword in Gaussian 16). The convergence of the geometry optimization is confirmed by the absence of imaginary frequencies within the harmonic approximation. Natural transition orbitals (NTOs) are computed with the TheoDORE program package[12] and visualized using Jmol[13].

Computation of vibrationally-resolved spectra

Vibrationally-resolved spectra are computed using the Franck–Condon Herzberg–Teller method developed by Barone and coworkers[14, 15] and implemented in Gaussian 16, revision C.01[5]. This method computes the nuclear wavefunctions within the harmonic approximation as well as the change of TDMs with respect to the normal coordinates of the molecule in a first order approximation to account for symmetry-allowed and symmetry-forbidden transitions. For this, an estimation of the potential energy surface (PES) in the harmonic approximation is needed, which is generated by performing a frequency calculation on the optimized minimum structures of the states involved in the transition. For the excitation from the ground state to the S_1, the optimized ground state and the optimized first singlet excited state structures were used. For the excitation to the S_4, we used the optimized ground state structure and also optimized the minimum of the emissive state in the donor, which is the S_4 at the Franck-Condon region (as referred to in the main manuscript), but actually the S_3 at its equilibrium geometry. For the sake of clarity, we refer to this state as the S_4 both in the main manuscript as well as here in the SI, even though it is not always a strictly energetic labelling. The spectra are convoluted from the vertical transitions using Gaussian functions of a full width at half maximum of 0.08 eV.

S2 MD Simulation Protocol

The MD simulations are performed using the program packages Amber20 and AmberTools21[16]. BPDI-PDI is described using the Generalized Amber Force Field (GAFF)[17] included in AmberTools21. The electrostatic potential for BPDI-PDI is computed using the DFT level of theory described above and fitted to the nuclei using the restrained electrostatic potential atomic partial charges (RESP) scheme using the antechamber program included in AmberTools21. Initial conditions are generated by embedding the solute in a truncated octahedral box of chloroform

molecules described using the force field parameters provided by AmberTools21. The size of the box is chosen such that every atom of the solute is at least 20 Å apart from any border of the box. The system is minimized using the Amber module pmemd in 5,000 minimization cycles employing a steepest descent algorithm, and another 5,000 additional steps using a conjugate gradient algorithm.

For all MD simulations, a time step of 2 fs is used. This large time step is enabled by turning on the SHAKE algorithm[18], which freezes hydrogen bond lengths at a relative geometrical tolerance of 1e-7. Constant pressure periodic boundary conditions are superimposed with isotropic pressure scaling. The cutoff for non-bonded interaction terms is set to 10 Å. The simulation is performed using the GPU (CUDA) version of pmemd[19–21].

The minimized system is subsequently heated using the Langevin thermostat at a collision frequency of 1.0 ps^{-1} to 100 K in 2,500 time steps (5 ps), followed by a heating to 300 K in 50,000 steps (100 ps). The first heating is performed at a pressure relaxation time of 1 ps, which is increased to 2 ps for the second heating phase only. The analysis of the simulation trajectories is performed using the program CPPTRAJ[22]. Visualization is done using the molecular viewer VMD[23].

S3 Role of Intermolecular Energy Transfer

In order to elucidate the possibility of intermolecular RET between different BPDI-PDI molecules, two dyads are placed into a chloroform box in close proximity (Figure S1).

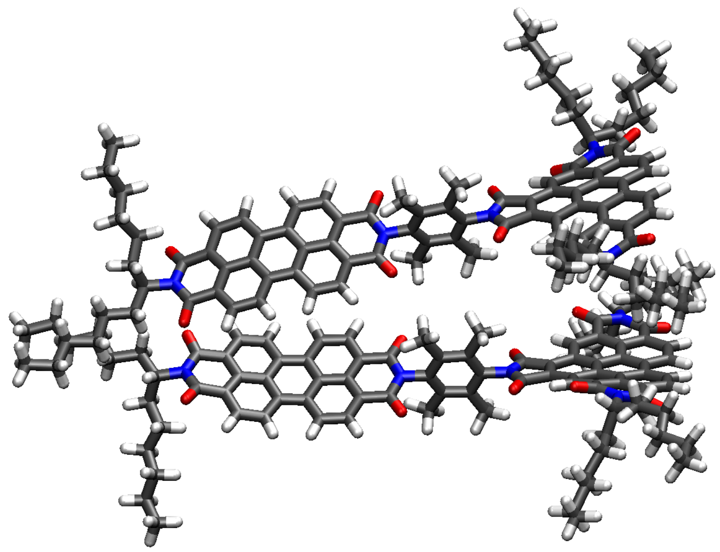

Figure S1: Starting structure of the simulation with two full BPDI-PDI molecules.

The system is propagated for 280 ns production according to the protocol described above. For sake of referencing, the dyads are randomly labelled 1 and 2. Consequently, the donor fragment in molecule 1 is labelled D_1, the acceptor in molecule 1 A_1, and the respective fragments in molecule 2 are labelled as D_2 and A_2. During the simulation, the center-of-mass distance

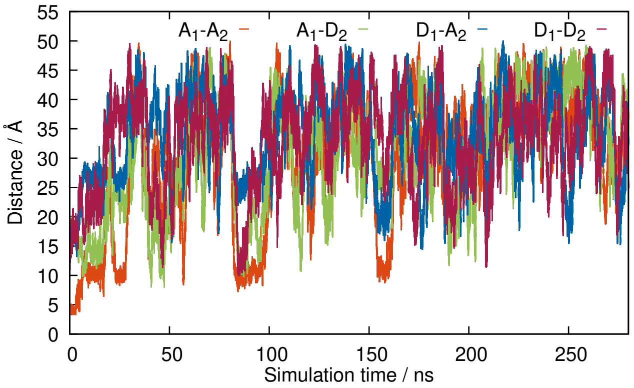

Figure S2: Center of mass distance between the acceptor (A) and donor (D) fragments in molecule 1 and 2 (denoted as subscripts).

between intermolecular fragments (A_1-A_2, A_1-D_2, D_1-A_2, D_1-D_2) is tracked.

As seen in Figure S2, the molecules move apart within the first 25 ns, with distances quickly exceeding 40 Å. As the periodic box has a side length of about 80 Å, this is the furthest distance apart possible between the molecules. At around 90-100 ns as well as after 150 ns, the molecules appear to come closer again, but center-of-mass distances rarely fall short of 10 Å. On the whole, there is no attractive interaction visible between the two molecules. In general, the distances between the fragments are so large, that, even though intermolecular energy transfer can not be entirely ruled out, its contribution seems irrelevant compared to the efficient intramolecular energy transfer, computed in the main text to occur in the picosecond regime.

S4 Computational Details at the Thermal Ensemble

Overview

In order to compute the electrostatic interaction factor $|V_{\mathrm{DA}}|^2$ for the thermal ensemble, we employed a hybrid QM/MM (quantum mechanics/molecular mechanics) approach, as described below. First, we generate 100 structures from a 1 ns MM-MD simulation carried out with Amber20. Then, these MM geometries are relaxed by performing a QM/MM-MD propagation during a random time between 150-200 fs, such that not all the geometrical parameters coherently converge to the same structures. In order to simulate the dyad in its appropriate electronic state in the QM/MM simulations, we first perform a ground state propagation of the entire dyad, which serves as a reference and justification for partitioning the system into individual QM regions for the donor and acceptor molecule. Then, the acceptor is described in the electronic ground state, and the donor in the electronic S_1 excited state. The environment is described within the classical MM approach using electrostatic embedding. We perform two dynamics simulations for each structure, on of the donor region (in the excited state), one of the acceptor region (in the ground state). The simulation is performed with Amber20 and Terachem[24–26]. Finally, using randomized QM/MM snapshots of BPDI-PDI, single point TD-DFT calculations at the B3LYP/def2-SVP level of theory as implemented in Gaussian 16[5] are

performed to yield the TDMs. As above, the environment is represented by point charges in these calculations. Below we provide details on each of these computational steps.

QM/MM equilibration of the entire dyad

Our first step is to perform MD simulations of the truncated BPDI-PDI dyad (see Figure 1 of the main text) in chloroform, according to the simulation protocol described in Section S2. The system is propagated during 1 ns in a single trajectory, from which snapshots are taken every 10 ps to yield 100 structures. On each of these 100 structures, we carry out a QM/MM-MD simulation at the B3LYP/def2-SVP level of theory in order to relax the bond lengths and angles from the inferior force field geometries to the more accurate DFT geometries.

In order to properly compute the energy transfer, the dyad needs to be propagated in its electronic state directly prior to the transfer. Usually, this is the minimum energy geometry of the energy donating state (S_4 in the *frozen* equilibrium structure). However, during dynamics simulations, it is quite common that the electronic states switch their energetic order due to geometrical distortions, so that a state of one character is, for instance, the fourth-lowest in energy in a particular time step, but might be third-lowest or fifth-lowest in the next time step. This behavior has been observed already at the S_4 optimization step (which finally was optimized as the S_3, see Section S1) and it was also present during the simulations. Therefore, we opt for an alternative approach, i.e. we partition the molecule into two separate QM regions, one for the acceptor fragment, another for the donor fragment. This simplifies the selection of the correct state, as outlined below. Naturally, such a partitioning is only possible assuming there is no wave function delocalization of one excitation over more than one fragment at a time. That there is no wave function overlap for the bright, low-energy excitations in the *frozen* equilibirum is evidenced by the NTOs shown in Figure 2b of the main text. Here we show that this is also the case for the full ensemble; therefore, we propagate the entire dyad in the QM region in its electronic ground state at the B3LYP/def2-SVP level of theory, as implemented in Terachem, version 1.9.2018.07-dev[24–26]. Point charges from the solvent atoms are included in the quantum chemical Hamiltonian up to a distance of 8 Å from any atom in the QM region. The solvent molecules are propagated using MM. We propagate each of the 100 structures for a random time between 150 and 200 ns on the QM/MM level. The simulation time is chosen at random within this time frame in order to avoid all the geometrical parameters within the ensemble to coherently converge to the same values while relaxing from the force field to the QM level of theory.

Out of the 100 structures, 96 were successfully propagated for the targeted time. The remaining four trajectories, where the simulation did not converge during some time step, were excluded from the analysis. On each of the final 96 geometries, we computed 30 singlet excited states with TD-DFT on the B3LYP/def2-SVP level of theory in Gaussian 16, again including point charges up to a distance of 8 Å.

Investigation of the electron localization

We analyzed the transition density matrix of the resulting 2,880 excitations using the TheoDORE package[12]. We computed to what fraction the excited electron is localized on the donor and acceptor fragments, irrespective of where the hole is located. This analysis serves to justify the partitioning into individual QM regions. The partitioning into donor and acceptor corresponds to what is shown in Figure 1 of the main text, with the linker described as a third fragment.

Figure S3 shows the amount of localization of the excited electron on the donor fragment plotted against that on the acceptor fragment. This representation shows the counteracting nature of the localizations: Whenever the localization is high on the donor fragment, it is small on the acceptor, and *vice versa*. The trend is very close to linear, which shows that in none of the 2,880 states, the excited electron is localized on the linker fragment. Instead, in most of the states, the excited electron is localized one either the donor or the acceptor fragment. This can be seen better from the histogram in Figure S3 that plots the number of points within a 1%-bin for the localization on the acceptor. About 1,000 are almost fully (> 99.5%) localized on the acceptor, and almost 1,600 of the excitations are not at all (< 0.5%) localized on the acceptor, thus mostly localized on the donor. In fact, only 30 excited electronic wave functions are not localized by at least 90% on either fragment. This analysis justifies that, due to the exclusive localization of excitations on only one fragment, it is reasonable to partition the dyad into individual regions, without loosing the main character of the electronic excitations.

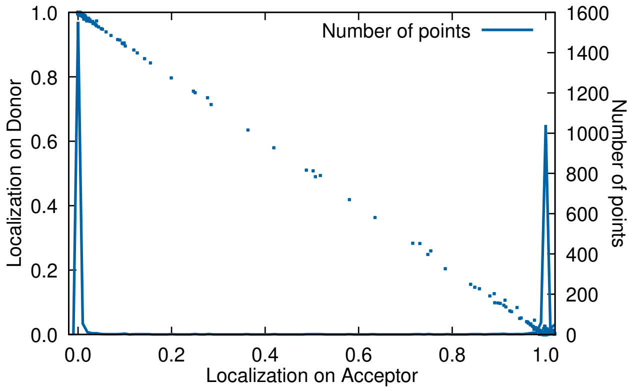

Figure S3: Fraction of localization of the excited state electron on the acceptor and donor fragments shown as points. A histogram of the number of points in intervals of 1% is overlaid as a line.

QM/MM equilibration of the individual regions

We justified the partition of the dyad into two QM regions. However, simulations with two QM regions cannot be performed with Amber20[16]. Thus, we adopt the following procedure in which for each of the 100 structures obtained from the MM-MD simulation, two QM/MM trajectories are simulated: one with the donor in the QM region and another with the acceptor in the QM region. Thus, in total, we perform 200 QM/MM simulations, half of which in the excited state. In both cases, the respective QM region is described at the B3LYP/def2-SVP level of theory

and executed with Terachem, version 1.9.2018.07-dev[24–26]. In each case, the remainder of the system, i.e. the rest of the dyad (including the linker), as well as the solvent molecules, are placed in the MM region. Whenever a covalent bond crosses the border between regions, the link atom approach implemented in Amber20 is used. Each of the two paired trajectories are computed at the QM/MM level for the same random time between 150 and 200 ns.

As stated above, the QM/MM propagations require to choose an electronic state in which to propagate the respective QM region. For the acceptor fragment, this choice is quite straightforward, as the acceptor is in the ground state before the energy is transferred. Thus, we propagate the acceptor in the electronic ground state. The donor is in the excited state, and the selected state needs to correspond to the S_4 *in the dyad*, as this is the state we identified as the donating state. As the S_4 in the dyad is the energetically lowest excited state, where both hole and electron are localized on the donor fragment, this corresponds to the S_1 excited state, when only the donor is in the QM region. Setting the electronic excited state of the donor region to S_1 implies that the propagation will occur in a thermal ensemble around the S_1 minimum. This is in line with Kasha's rule[27], which says that any excited state populations relax to the lowest excited state of the respective multiplicity prior to emission of radiation. Very efficient energy transfers might not totally abide to that rule, if they are fast enough to already occur close to the Franck-Condon region. However, while the energy transfer computed in the main text occurs within the picosecond time scale, vibrational relaxation usually occurs in the femtosecond regime[28]. Judging from this difference in time scales, the influence of energy transfer prior to vibrational relaxation should be insignificant. Additionally, geometrical changes between the Franck-Condon region and the excited state minimum are, in general, not very drastic in aromatic molecules, so that we do not have to expect significantly altered energy transfer efficiencies at the different geometries. Accordingly, propagation in the S_1 minimum of the donor QM region should accurately describe all properties relevant for the energy transfer rate.

Validity and applicability of the approach

In order to ensure that the properties, especially the TDMs, are still comparable between the two paired trajectories, we contrast their corresponding structures and compute the heavy-atom (excluding hydrogens) root mean squared deviation (RMSD, see Figure S4a).

As it can be seen, the bulk of structure pairs exhibit RMSD's between 0.10 and 0.15 Å, with the strongest-diverging structures at 0.19 Å. The two structures with the largest structural difference are exemplary shown in Figure S4b. There are some small displacements of individual atoms, which can have an impact on the TD-DFT results, such as on the energy gap between ground and excited states, as well as on the magnitude and directionality of TDMs. These small displacements between the force field geometries and those obtained from a propagation with DFT is the reason why we propagate each region with quantum mechanics before computing excited state properties. However, aside from these small displacements, the general shape and orientation of the fragments is not different, such that TDM orientations computed on the accurate, QM geometry can be translated onto the respective fragment propagated with MM.

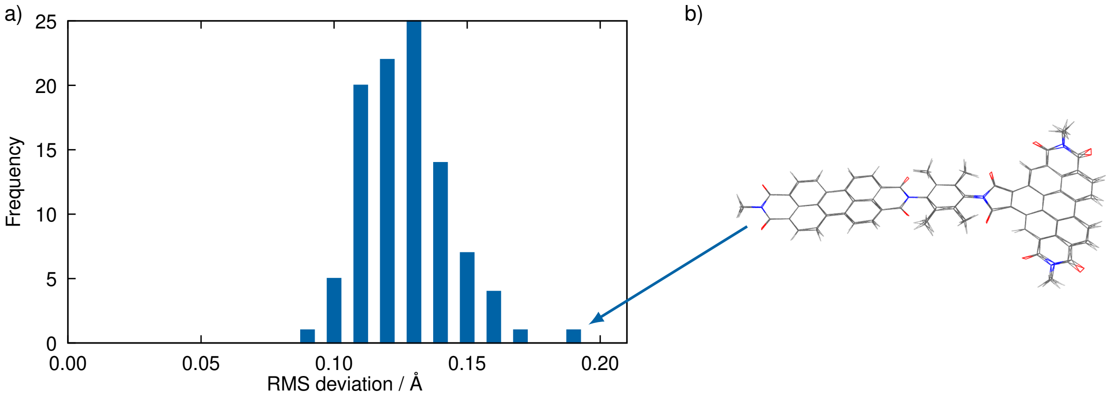

Figure S4: a) Heavy-atom RMSD of BPDI-PDI for the 100 pairs of diverging trajectories of different QM regions. b) Pair of structures with the highest RMSD.

Finally, we used the obtained 100 pairs of BPDI-PDI structures and all chloroform point charges within 8 Å to any solute atom to perform TD-DFT single point calculation on both of the QM regions individually using the Gaussian 16[5] protocol (Section S1) to compute the TDMs, their magnitude and orientation, and used the result to compute the electrostatic interaction factor $|V_{\mathrm{DA}}|^2$.

S5 Character of the excitations in the ensemble

Investigation of the NTOs

In the main text, it was claimed that whenever the angle θ_{D} between $\vec{\mu}_{\mathrm{D}}$ and the N_4-N_5 vector approaches orthogonality, the charge transfer character of the excitation is increased. The NTOs for two structures representative of this behaviour are shown in Figure 4 of the main manuscript. These structures exhibit the most extreme values of θ_{D} found in the entire ensemble of 100 structures. The structure shown in the left box has the lowest θ_{D} at around 0.7°, and the structure on the right has the highest θ_{D} of around 89.9°.

Both structures have the electron and the hole in the same two corresponding orbitals, albeit with different contributions. For both structures, the hole is composed of two π orbitals localized over the aromatic rings, one symmetric with respect to the mirror plane perpendicular on the molecular plane, the other antisymmetric. The excited electrons are located in an orbital described as the linear combination of two orbitals, one delocalized over most of the aromatic system, as well, but the other is mostly localized on the five-membered ring. However, the combination of electron and hole between the two structures are swapped: For the structure, where θ_{D} is close to 0°, excitation is transferred from the antisymmetric hole to the mostly delocalized electron, and from the symmetric hole to the electron localized mostly on the five-membered ring. In the other structure with the orthogonal TDM, the antisymmetric hole is paired with the localized electron, and consequently the symmetric hole with the delocalized electron. Furthermore, the weights of the transitions are different. In the structure with the

parallel TDM, 95% of the excitation are between the orbitals which are delocalized over most of the fragment, and only 5% are associated with a charge transfer to the five-membered ring. This trend is flipped in the structure, where θ_D is close to 90°. Here, 82% of the excitation are associated with the charge transfer. This increased charge transfer character is responsible for the altered direction of the TDM, but also the reduction in intensity as discussed in the main text. Here, the structure with the TDM aligned parallel exhibits a TDM strength of 6.7 au, while it is only 0.5 au in the structure with the orthogonal TDM.

Comparison between the donor fragment propagated in the ground state and in the excited state

This increased CT character and, with it, the increased θ_D angle, is a feature of the ensemble equilibrated in the excited state. This can be seen in Figure S5, where the θ_D, the angle between the TDM on the donor fragment and the N_4-N_5 vector is shown for the donor equilibrated in the ground state (Figure S5 left) and after equilibration in the excited state (Figure S5 right). The

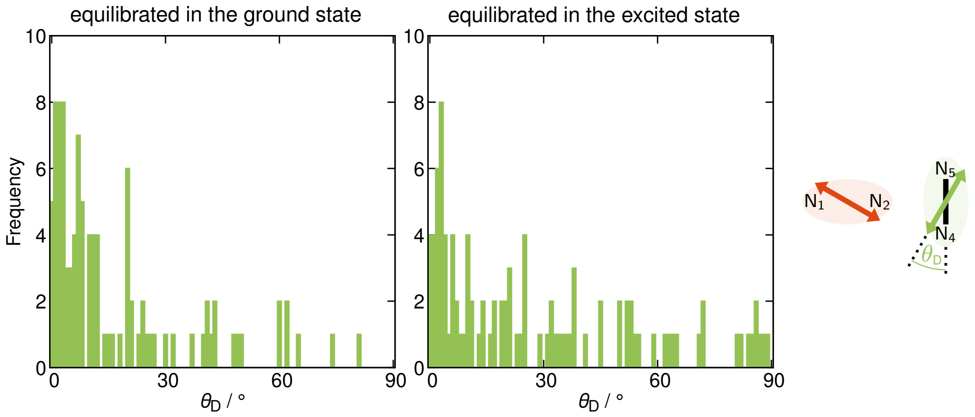

Figure S5: Histograms of the angle θ_D for the thermal ensemble equilibrated in the ground (left plot) and the excited state (right plot). The data depicted in the right plot is identical to that of Figure 3d of the main manuscript. The pictogram on the right is an illustration of the angle's geometry.

overall distribution of the angles is comparable between the two. However, in the ground state ensemble, more angles are close to 0°, while the excited state ensemble exhibits more TDMs close to orthogonal to the N_4-N_5 vector. This implies, that the CT character of the donating state increases upon relaxation towards the excited state minimum. Unfortunately, since in our approach we do not simulate proper excited state relaxation dynamics, but merely switch the state of interest in the QM region from ground state to excited state, our data does not give insights into the time scales on which this character is altered during excited state relaxation.

S6 Details on the RET rate computation

For each of the 100 structures in the thermal ensemble, each represented as two QM/MM structures pairs (Section S5), we compute the electrostatic interaction factor $|V_{\mathrm{DA}}|^2$. For this, we need to compute the distance between the donor and the acceptor fragment. Again, we use the definition for the fragment centers as the midpoint of the respective N-N connecting vector as described above. At these short intramolecular distances and with frontier orbitals delocalized over the entire π manifolds of the respective fragments, these centers are not an ideal description of the *de facto* interaction centers. First, the interaction between electron densities in closer proximity will most certainly exceed those of further apart fractions. At larger distances, where the total distance is much bigger than the diameter of the fragments, this effect is negligible, but at the comparatively short distances we are dealing with here, a point-dipole interaction on a nucleus-nucleus basis could provide more accurate results. Second, our definition of a fragment center is not equivalent to other definitions, such as the center of mass. This is most notably the case for the donor fragment, where, due to its reduced symmetry compared to the acceptor fragment, the center of mass is shifted closer to the acceptor than the midpoint between N_4 and N_5 is. Nevertheless, we deem this N-N connecting vector approach very intuitive and easy to interpret, and believe that due to likely cancellation of the described errors, this is still a suitable representation. For computing $|V_{\mathrm{DA}}|^2$, we used a refractive index η of 1.44 for chloroform in the visible region[29].

With $|V_{\mathrm{DA}}|^2$ and J, the RET rate can be straightforwardly computed as,

$$k_{\mathrm{RET}} = \frac{1}{\hbar^2 c}|V_{\mathrm{DA}}|^2 J = \frac{1}{\hbar^2 c} * 5.2 \times 10^{-44}\mathrm{J}^2 * 7.44 \times 10^{-5}\mathrm{m} = 1.2 \times 10^{12}\mathrm{s}. \tag{1}$$

References

(1) J. P. Perdew, *Phys. Rev. B*, 1986, **33**, 8822–8824.

(2) C. Lee, W. Yang and R. G. Parr, *Phys. Rev. B*, 1988, **37**, 785–789.

(3) F. Weigend and R. Ahlrichs, *Phys. Chem. Chem. Phys.*, 2005, **7**, 3297.

(4) F. Weigend, *Phys. Chem. Chem. Phys.*, 2006, **8**, 1057.

(5) M. J. Frisch, G. W. Trucks, H. B. Schlegel, G. E. Scuseria, M. A. Robb, J. R. Cheeseman, G. Scalmani, V. Barone, G. A. Petersson, H. Nakatsuji, X. Li, M. Caricato, A. V. Marenich, J. Bloino, B. G. Janesko, R. Gomperts, B. Mennucci, H. P. Hratchian, J. V. Ortiz, A. F. Izmaylov, J. L. Sonnenberg, D. Williams-Young, F. Ding, F. Lipparini, F. Egidi, J. Goings, B. Peng, A. Petrone, T. Henderson, D. Ranasinghe, V. G. Zakrzewski, J. Gao, N. Rega, G. Zheng, W. Liang, M. Hada, M. Ehara, K. Toyota, R. Fukuda, J. Hasegawa, M. Ishida, T. Nakajima, Y. Honda, O. Kitao, H. Nakai, T. Vreven, K. Throssell, J. A. Montgomery Jr., J. E. Peralta, F. Ogliaro, M. J. Bearpark, J. J. Heyd, E. N. Brothers, K. N. Kudin, V. N. Staroverov, T. A. Keith, R. Kobayashi, J. Normand, K. Raghavachari, A. P. Rendell, J. C. Burant, S. S. Iyengar, J. Tomasi, M. Cossi, J. M. Millam, M. Klene,

C. Adamo, R. Cammi, J. W. Ochterski, R. L. Martin, K. Morokuma, O. Farkas, J. B. Foresman and D. J. Fox, *Gaussian16 Revision C.01*, 2016.

(6) H. Langhals, S. Poxleitner, O. Krotz, T. Pust and A. Walter, *Eur. J. Org. Chem.*, 2008, **2008**, 4559–4562.

(7) H. Langhals, A. J. Esterbauer, A. Walter, E. Riedle and I. Pugliesi, *J. Am. Chem. Soc.*, 2010, **132**, 16777–16782.

(8) V. Barone and M. Cossi, *J. Phys. Chem. A*, 1998, **102**, 1995–2001.

(9) M. Cossi, N. Rega, G. Scalmani and V. Barone, *J. Comput. Chem.*, 2003, **24**, 669–681.

(10) S. Grimme, J. Antony, S. Ehrlich and H. Krieg, *J. Chem. Phys.*, 2010, **132**, 154104.

(11) S. Grimme, S. Ehrlich and L. Goerigk, *J. Comput. Chem.*, 2011, **32**, 1456–1465.

(12) F. Plasser, *J. Chem. Phys.*, 2020, **152**, 084108.

(13) Jmol development team, *Jmol*, version 14.6.4, 2016.

(14) F. Santoro, A. Lami, R. Improta, J. Bloino and V. Barone, *J. Chem. Phys.*, 2008, **128**, 224311.

(15) V. Barone, J. Bloino, M. Biczysko and F. Santoro, *J. Chem. Theory Comput.*, 2009, **5**, 540–554.

(16) D. Case, H. Aktulga, K. Belfon, I. Ben-Shalom, S. Brozell, D. Cerutti, T. Cheatham, Ill, V. Cruzeiro, T. Darden, R. Duke, G. Giambasu, M. Gilson, H. Gohlke, A. Goetz, R. Harris, S. Izadi, S. Izmailov, C. Jin, K. Kasavajhala, M. Kaymak, E. King, A. Kovalenko, T. Kurtzman, T. Lee, S. LeGrand, P. Li, C. Lin, J. Liu, T. Luchko, R. Luo, M. Machado, V. Man, M. Manathunga, K. Merz, Y. Miao, O. Mikhailovskii, G. Monard, H. Nguyen, K. O'Hearn, A. Onufriev, F. Pan, S. Pantano, R. Qi, A. Rahnamoun, D. Roe, A. Roitberg, C. Sagui, S. Schott-Verdugo, J. Shen, C. Simmerling, N. Skrynnikov, J. Smith, J. Swails, R. Walker, J. Wang, H. Wei, R. Wolf, X. Wu, Y. Xue, D. York, S. Zhao and P. Kollman, *Amber 2021*, San Fransisco, 2021.

(17) J. Wang, R. M. Wolf, J. W. Caldwell, P. A. Kollman and D. A. Case, *J. Comput. Chem.*, 2004, **25**, 1157–1174.

(18) J.-P. Ryckaert, G. Ciccotti and H. J. Berendsen, *J. Comput. Phys.*, 1977, **23**, 327–341.

(19) R. Salomon-Ferrer, A. W. Götz, D. Poole, S. L. Grand and R. C. Walker, *J. Chem. Theory Comput.*, 2013, **9**, 3878–3888.

(20) A. W. Götz, M. J. Williamson, D. Xu, D. Poole, S. L. Grand and R. C. Walker, *J. Chem. Theory Comput.*, 2012, **8**, 1542–1555.

(21) S. L. Grand, A. W. Götz and R. C. Walker, *Comput. Phys. Commun.*, 2013, **184**, 374–380.

(22) D. R. Roe and T. E. Cheatham, *J. Chem. Theory Comput.*, 2013, **9**, 3084–3095.

(23) W. Humphrey, A. Dalke and K. Schulten, *J. Mol. Graphics*, 1996, **14**, 33–38.

(24) I. S. Ufimtsev and T. J. Martinez, *J. Chem. Theory Comput.*, 2009, **5**, 2619–2628.

(25) A. V. Titov, I. S. Ufimtsev, N. Luehr and T. J. Martinez, *J. Chem. Theory Comput.*, 2012, **9**, 213–221.

(26) S. Tomov, J. Dongarra and M. Baboulin, *Parallel Comput.*, 2010, **36**, 232–240.

(27) M. Kasha, *Discuss. Faraday Soc.*, 1950, **9**, 14.

(28) P. Klán and J. Wirz, in ed. P. Klán and J. Wirz, Wiley, 2009, ch. A Crash Course in Photophysics and a Classification of Primary Photoreactions, pp. 25–72.

(29) S. Kedenburg, M. Vieweg, T. Gissibl and H. Giessen, *Opt. Mater. Express*, 2012, **2**, 1588.
